# Combination of an Optically Induced Dielectrophoresis (ODEP) Mechanism and a Laminar Flow Pattern in a Microfluidic System for the Continuous Size-Based Sorting and Separation of Microparticles

**DOI:** 10.3390/bios14060297

**Published:** 2024-06-06

**Authors:** Po-Yu Chu, Ai-Yun Wu, Kun-Yu Tsai, Chia-Hsun Hsieh, Min-Hsien Wu

**Affiliations:** 1 Graduate Institute of Biomedical Engineering, Chang Gung University, Taoyuan City 33302, Taiwan; 2 Division of Colon and Rectal Surgery, New Taipei Municipal TuCheng Hospital, New Taipei City 23652, Taiwan; 3 Division of Hematology/Oncology, Department of Internal Medicine, Chang Gung Memorial Hospital at Linkou, Taoyuan City 33302, Taiwan; 4 Division of Hematology/Oncology, Department of Internal Medicine, New Taipei Municipal Hospital, New Taipei City 23652, Taiwan; 5 College of Medicine, Chang Gung University, Taoyuan City 33302, Taiwan; 6 Department of Biomedical Engineering, Chang Gung University, Taoyuan City 33302, Taiwan

**Keywords:** microfluidic technology, optically induced dielectrophoresis (ODEP), laminar flows, microparticles, size sorting

## Abstract

Optically induced dielectrophoresis (ODEP)-based microparticle sorting and separation is regarded as promising. However, current methods normally lack the downstream process for the transportation and collection of separated microparticles, which could limit its applications. To address this issue, an ODEP microfluidic chip encompassing three microchannels that join only at the central part of the microchannels (i.e., the working zone) was designed. During operation, three laminar flows were generated in the zone, where two dynamic light bar arrays were designed to sort and separate PS (polystyrene) microbeads of different sizes in a continuous manner. The separated PS microbeads were then continuously transported in laminar flows in a partition manner for the final collection. The results revealed that the method was capable of sorting and separating PS microbeads in a high-purity manner (e.g., the microbead purity values were 89.9 ± 3.7, 88.0 ± 2.5, and 92.8 ± 6.5% for the 5.8, 10.8, and 15.8 μm microbeads harvested, respectively). Overall, this study demonstrated the use of laminar flow and ODEP to achieve size-based sorting, separation, and collection of microparticles in a continuous and high-performance manner. Apart from the demonstration, this method can also be utilized for size-based sorting and the separation of other biological or nonbiological microparticles.

## 1. Introduction

The sorting and separation of natural microparticles, synthetic microparticles, or biological substances at the microscale (e.g., cells or bacteria) is a fundamental step for subsequent analysis or applications [1,2]. With the advances in separation and purification technologies, size-based sorting and separation of microparticles can be achieved through centrifugation or membrane separation [1,2]. Although these mature technologies have been successfully implemented in industrial or academic settings, these conventional technologies are not suitable when the sample is limited or when high-purity sorting and separation of microparticles are needed; this is mainly due to their inappropriate dimensional scale or technical limitations. The proper dimensional feature of a microfluidic system is suitable for addressing these tasks, as mentioned above.

With the recent progress in microfluidic technology, microfluidic systems with different designs have been developed to sort, separate, and purify microparticles based on their size differences [3,4,5]. In general, the working mechanisms involve the use of microstructures [6,7], fluidic control [8,9,10,11], or external forces [12,13] to achieve these tasks. Several microfluidic systems utilize microstructures with various geometries that mimic the pores in a membrane for the size-based sorting and separation of microparticles [14,15]. Although most of these methods have been proven to be feasible, the possible technical problems relevant to microparticle clogging, which could in turn affect function, have not been well discussed. Moreover, it is generally believed that the fabrication of microstructures is normally costly and technically demanding, which might hinder their widespread application. Conversely, microfluidic systems utilizing fluidic manipulation for the sorting and separation of microparticles could largely reduce the requirements of microfabrication. For example, several studies (e.g., deterministic lateral displacement- [6,7], inertial microfluidics- [8,9], or viscoelastic microfluidics [10,11]-based methods) have successfully demonstrated the ability of these methods to effectively sort and separate microparticles based on their size differences. However, the delicate manipulation of microflows in a microfluidic system is still technically demanding.

In addition to the two abovementioned mechanisms, the incorporation of external energy [e.g., acoustophoresis- [16,17], magnetophoresis- [18,19], dielectrophoresis (DEP)- [20,21,22], or optically induced dielectrophoresis (ODEP) [23,24]-based force] in microfluidic systems has been presented for the effective sorting and separation of microparticles for various purposes. Among these techniques, the use of DEP has attracted the interest of scientists due to its ability to manipulate microparticles well. The working principle of DEP-based microparticle manipulation has been well described elsewhere [20] and is briefly described herein. When a dielectric microparticle is placed in an electric field, the microparticle can be electrically polarized. The microparticle is further subjected to a localized electric field. The interaction between the induced charges on the electrically polarized microparticle and the electric field exerted around the microparticle can generate a force called the DEP force [20]. The DEP force can then drive the movement of microparticles. Based on this phenomenon, microparticles can be manipulated in a controllable manner via manipulation of the electric field around the microparticles (e.g., the use of a microelectrode array for this purpose [20]). In addition to microparticle manipulation, the DEP force generated on a microparticle is proportional to the cube of its radius [20,21,22]. Therefore, DEP force-based microparticle manipulation can also be utilized for the size-based sorting and separation of microparticles, which has been well demonstrated elsewhere [20,21,22]. Nevertheless, DEP force-based microparticle manipulation commonly requires technically demanding and costly microfabrication to create a metal microelectrode array for a specific application [13,21,25,26], which might affect its practical applications.

Compared with the DEP-based technique, the ODEP-based technique, first presented in 2005 [25], can use light images, serving as virtual electrodes to replace the microelectrodes in the DEP setting, for microparticle manipulation. This technical feature not only eliminates the need for microfabrication but also allows the users to adjust the virtual electrode design in an easy and flexible manner simply via the modification of light images in an ODEP system. The working principle of ODEP-based microparticle manipulation, similar to that of DEP manipulation, is well described elsewhere [23,24,25,26]. Briefly, an alternating current (AC) voltage is applied between the top and bottom substrates of an ODEP system, in which a thin solution layer containing microparticles is sandwiched between the substrates; this causes the microparticles in the solution to be electrically polarized. When the bottom layer (i.e., a photoconductive layer) is illuminated with light, the voltage can decrease in the light-illuminated area. Based on this phenomenon, a locally nonuniform electric field in the light-illuminated region is generated. In ODEP force-based microparticle manipulation, the interaction between a light-induced nonuniform electric field and an electrically polarized microparticle is used to manipulate the microparticle. Overall, one can simply use dynamic or stationary optical images that are illuminated on the photoconductive layer to manipulate microparticles in a manageable manner. Reports in the literature have demonstrated the utilization of the ODEP mechanism for various applications, including the manipulation of magnetic microparticles for biosensing [27], the sorting and separation of cells with varied degrees of cell viability [28], the isolation and purification of cells in samples [29], and the sorting and separation of PS (polystyrene) microbeads of different sizes [26,30].

Among the ODEP applications, ODEP-based microparticle manipulation has been demonstrated to sort and separate microparticles of different sizes in a high-purity manner [26,30]; nevertheless, its overall process was based on a batchwise operation model, lacking the downstream operation process for the partitioning, transportation, and collection of the sorted and separated microparticles. Therefore, the proposed design could limit its applications for high-throughput or continuous size-based microparticle sorting and separation. To achieve both continuous and high-performance size-based microparticle sorting and separation, this study proposes the utilization of the laminar flow regime inherent in a microfluidic channel and ODEP-based microparticle manipulation for this purpose. To test the feasibility of the proposed method, an ODEP microfluidic chip encompassing three individual microchannels joining only at the central part of the microchannel was designed and fabricated. In the design, the junction zone of three microchannels was defined as the sorting and separation zone. During operation, fluid flows with equal volumetric flow rates were driven in the three individual microchannels to form three laminar flows in the sorting and separation zone. Moreover, two dynamic light bar arrays with different velocities were designed to sort and separate microparticles (i.e., the use of PS microbeads as a test model in this work) of three different sizes (i.e., 5.8, 10.8, and 15.8 μm in diameter) in a continuous-flow manner. The sorted and separated PS microbeads were then continuously transported in three laminar flows in a partial manner for the final collection purpose. Based on this design, PS microbeads of different sizes can be sorted, separated, transported, and collected in a continuous manner.

In this study, the optimal width (i.e., 75 μm) and velocity of motion of rectangular light bars in two dynamic light bar arrays (i.e., 50 and 95 μm s^−1^ for arrays I and II, respectively) for the size-based sorting and separation of PS microbeads were explored experimentally. Additionally, the optimal flow rate (i.e., 0.3 μL min^−1^) in the three microchannels was experimentally determined to ensure that the laminar microflows in the sorting and separation zone were stable. Moreover, the performance of the sorting and separation of PS microbeads was experimentally evaluated. The results revealed that the proposed method was capable of sorting and separating PS microbeads in a high-purity manner (e.g., the purities of the microbeads were 89.9 ± 3.7, 88.0 ± 2.5, and 92.8 ± 6.5% for the 5.8, 10.8, and 15.8 μm microbeads harvested, respectively). Overall, this study proposed utilizing the laminar flow pattern inherent in a microchannel and ODEP-based microparticle manipulation to achieve size-based sorting, separation, transport, and collection of microparticles in a simple, continuous, and high-performance manner. The proposed continuous operation scheme is not limited to the sorting and separation of the PS microbeads, as in this study; it can also be utilized for the sorting and separation of other biological or nonbiological microparticles based on their size differences.

## 2. Materials and Methods

### 2.1. The ODEP Microfluidic System for the Continuous Sorting and Separation of PS Microbeads of Different Sizes

**Figure 1:**
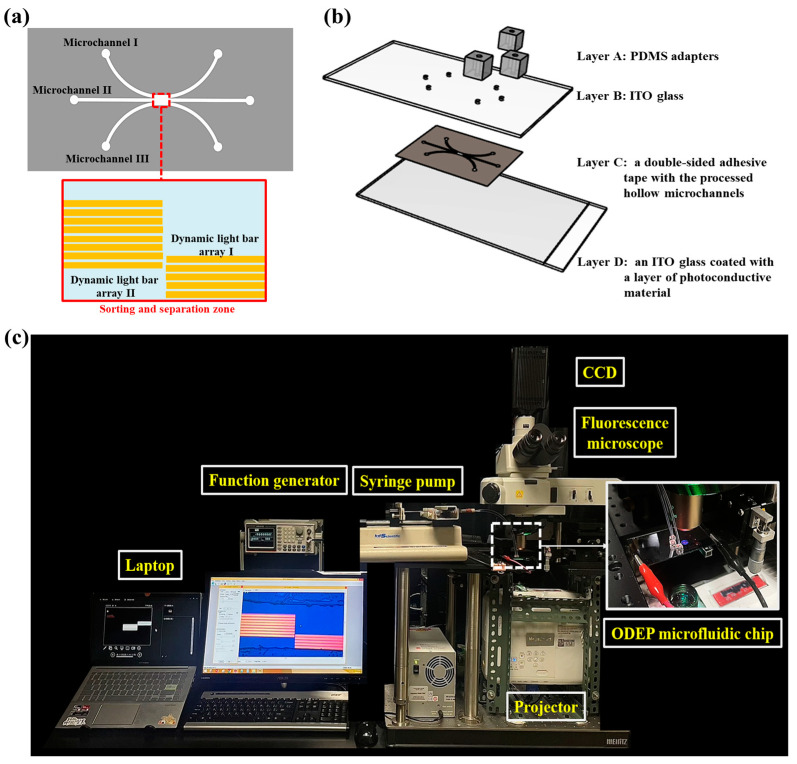
(**a**) Layout (top-side view), (**b**) structure (Layers A, B, C, and D: 3 PDMS tube adapters, ITO glass, double-sided adhesive tape with processed hollow microchannels, and ITO glass coated with a layer of photoconductive material, respectively), and (**c**) photograph of the overall experimental setup.

In this study, an ODEP microfluidic chip [top-side view layout; Figure 1a] was designed for the continuous sorting and separation of PS microbeads of three different sizes. Briefly, three individual microchannels [i.e., two U-shaped microchannels (L: 23.0 mm, W: 400 μm, H: 50.0 μm) and a middle straight microchannel (L: 21.6 mm, W: 400 μm, H: 50.0 μm)] were designed in the microfluidic chip for transporting the loaded PS microbead sample as well as the sorted and separated PS microbeads. In the design, the three microchannels join only at the central part of the microchannel, which is defined as the sorting and separation zone [L: 6.0 mm, W: 1.2 mm, H: 50.0 μm; Figure 1a], where two dynamic light bar arrays were designed to continuously sort and separate the loaded PS microbeads of three different sizes (i.e., the ODEP-based microparticle manipulation for the sorting and separation of microparticles). The structure of the ODEP microfluidic chip is schematically illustrated in Figure 1b. Briefly, the ODEP microfluidic chip mainly consisted of 3 tube adapters made of polydimethylsiloxane (PDMS) (Layer A), an indium tin oxide (ITO) glass with six holes (D: 1.0 mm) (Layer B), double-sided adhesive tape (thickness: 50 μm) with processed hollow microchannels (Layer C), and ITO glass coated with a layer of photoconductive material (Layer D).

The fabrication, assembly, and experimental setup of the ODEP microfluidic system were described in our previous studies [26,30] and are briefly discussed herein. Three tube adapters [Figure 1b] were fabricated by computer-numerical-controlled (CNC) machining for positive polymethylmethacrylate (PMMA) mold preparation and subsequent PDMS (Sylgard^®^ 184, Dow Corning, Midland, MI, USA) replica molding. For Layer B, six through-holes were mechanically drilled in ITO glass (R01, InnoLux Corporation, HSH, Miaoli City, Taiwan) using a drill. For Layer C, a hollow structure in double-sided adhesive tape (L298, Sun-yieh, Taoyuan City, Taiwan) was fabricated using laser cutting. For the bottom layer, Layer D, a 20 nm thick heavily n+-doped hydrogenated amorphous silicon (n+ a-Si:H) layer and a 1 μm thick amorphous silicon layer (a-Si:H) were deposited on the ITO glass via a PECVD process [26,30]. During the assembly process, Layer B was first assembled with Layer D through double-sided adhesive tape (i.e., Layer C). After that, the 3 tube adapters (i.e., Layer A) were bonded with Layer B with the aid of O2 plasma surface treatment.

In terms of operation, a suction-type multichannel syringe pump (KD Scientific, Holliston, MA, USA) was utilized to drive fluidic flow in the three microchannels. To achieve ODEP-based microparticle maintenance, a function generator was used to apply an alternating current (AC) between Layers B and D [Figure 1b]. A commercial digital projector (EB-X05, Epson, Nagano, Japan) coupled with a computer was used to display light images onto Layer D to generate ODEP force on the PS microbeads. Additionally, a CCD-equipped microscope (SPOT Insight Color, Diagnostic Instruments, Inc., Sterling Heights, MI, USA) was used to observe the manipulation of microbeads in the microfluidic chip. A photograph of the overall experimental setup is shown in Figure 1c.

### 2.2. The Designed ODEP-Based Mechanism for the Continuous Sorting and Separation of PS Microbeads Based on Their Size Differences

The working principle of ODEP-driven microparticle manipulation is described in the introduction section. The ODEP force acting on a microparticle can be expressed by Equation (1), which is also used to describe the DEP force [26,30]:

F_DEP_ = 2πr^3^ε_0_ε_m_Re[f_CM_]∇|E|^2^ (1)

where r, ε_0_, ε_m_, ∇|E|^2^, and Re[f_CM_] denote the microparticle radius, vacuum permittivity, relative permittivity of the surrounding solution, gradient of the exerted electrical voltage squared, and real part of the Clausius–Mossotti factor (f_CM_), respectively. According to the above equation, the ODEP force acting on a microparticle is proportional to its cubic radius under the given operation conditions (e.g., the electrical voltage or the property of the surrounding solution) [26,30]. Based on this phenomenon, two dynamic light bar arrays with different velocities were designed at the sorting and separation zone [Figure 1a] to sort and separate the PS microbeads based on their size differences in a continuous-flow manner.

**Figure 2:**
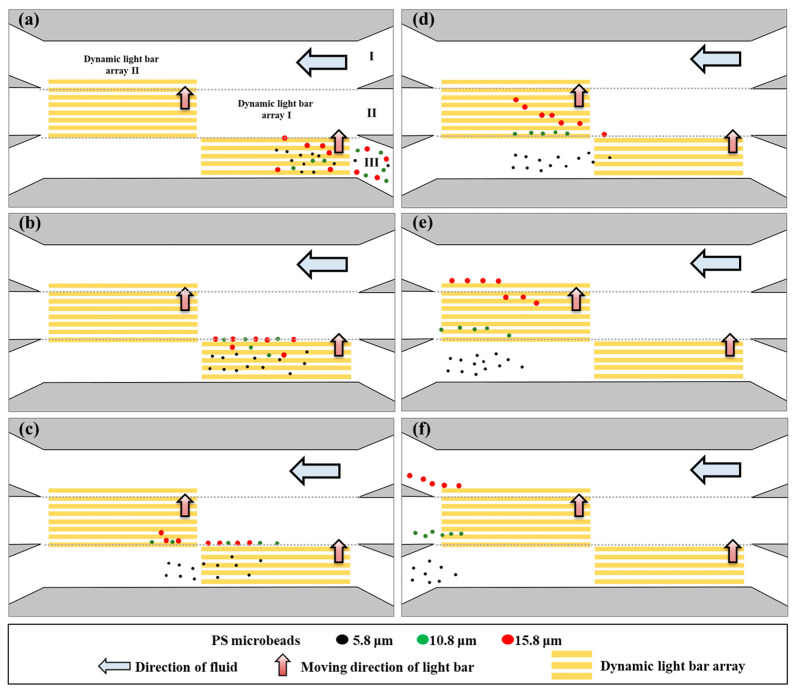
The overall working mechanism of the proposed method for the continuous size-based sorting and separation of PS microbeads: (**a**) the PS microbead sample with three different sizes was loaded and transported via microchannel III, (**b**) dynamic light bar array I at the inlet of microchannel III was used to sort and separate the 10.8 and 15.8 μm microbeads from the 5.8 μm microbeads, and (**c**) the separated 5.8 μm microbeads and the 10.8 and 15.8 μm microbeads were transported by the laminar flows in microchannels III and II, respectively. (**d**,**e**) Dynamic light bar array II located at the outlet of microchannel II was used to sort and separate the 10.8 and 15.8 μm microbeads. (**f**) The sorted and separated 10.8 and 15.8 μm microbeads were then transported by the laminar flows in microchannels II and I, respectively.

The overall working mechanism is schematically illustrated in Figure 2. Briefly, the working solution [0.05% (*w*/*v*) BSA in distilled water; conductivity: 6.5–8.0 μS cm^−1^] was individually transported in the three microchannels [i.e., microchannels I, II, and III of Figure 1a and Figure 2a] to form three separate laminar flows at the defined sorting and separation zone (i.e., the junction zone of the three microchannels). In this process, a PS microbead mixture of three different sizes (i.e., 5.8, 10.8, and 15.8 μm in diameter) (the optimal concentration range with less significant microbead aggregation: 200–300 microbeads per microliter) was loaded and transported via microchannel III, as illustrated in Figure 2a. In this work, dynamic light bar array I, encompassing 5 moving rectangular light bars [L: 1.8 mm, and W: 75 μm], was designed at the inlet of microchannel III. The PS microbeads that were 10.8 and 15.8 μm in diameter were sorted and separated from the 5.8 μm microbeads, as illustrated in Figure 2a,b. After that, the smallest microbeads (i.e., 5.8 μm) were transported by the laminar flow in microchannel III and finally collected via the outlet of microchannel III, as shown in Figure 2b–f. Larger separated microbeads (i.e., 10.8 and 15.8 μm) were then delivered forward via laminar flow in microchannel II [Figure 2b,c]. After reaching dynamic light bar array II (8 light bars; each light bar: L: 1.8 mm, and W: 75 μm) located at the outlet of microchannel II, the PS microbeads of two different sizes were then sorted and separated by the array based on their size differences, as shown in Figure 2c–e. The sorted and separated microbeads, 10.8 and 15.8 μm in diameter, were then transported by the laminar flows in microchannels II and I and finally collected via the outlets of microchannels II and I, respectively [Figure 2e,f].

### 2.3. The Operation Conditions for the Sorting and Separation of PS Microbeads

**Figure 3:**
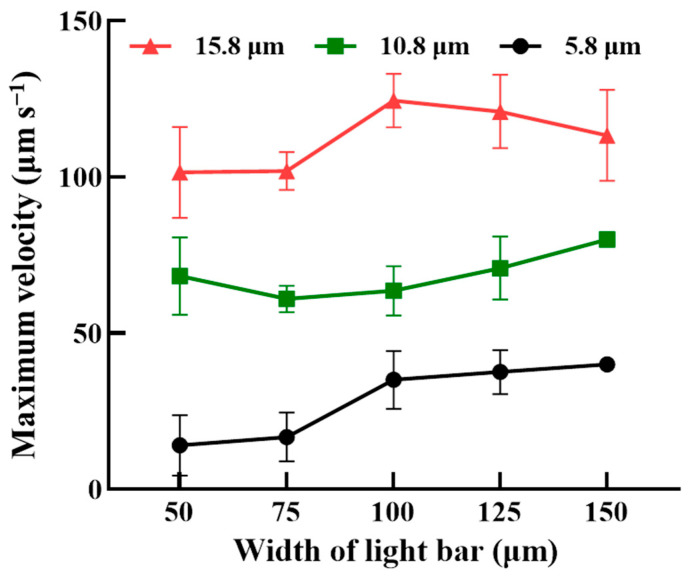
The maximum velocities of the rectangular light bar that could manipulate PS microbeads of three different sizes (i.e., 5.8, 10.8, and 15.8 μm in diameter) under various light bar widths (i.e., 50, 75, 100, 125, and 150 μm).

In this work, the basic ODEP operation conditions were set based on those in our previous work, such as electric voltage and frequency values of 10 Vpp and 1.5 MHz, respectively [30]. Moreover, for the operation scheme illustrated in Figure 3, the operation conditions, including the width of the light bars, the moving velocity of the light bars in arrays I and II, and the volumetric flow rate of the microchannels, were experimentally determined. In ODEP-based microparticle manipulation, the ODEP manipulation force acting on a manipulated microparticle can be experimentally evaluated based on a method described previously [26,30]. Briefly, the ODEP manipulation force acting on a manipulated microparticle is balanced by the viscous drag of the fluid. Therefore, the hydrodynamic drag force of a moving microparticle can be used to evaluate the ODEP manipulation force on the microparticle [26,30]. The hydrodynamic drag force (F) acting on a microparticle can be described by Stocke’s law (Equation (2) below) under flow conditions [26,30]:

F = 6πrηv(2)

In Equation (2), r, η, and v denote the radius of the microparticle, fluid viscosity, and maximum velocity of a moving microparticle, respectively. According to Equation (2), the ODEP manipulation force acting on a manipulated microparticle can be experimentally evaluated by measuring the maximum velocity of a dynamic optical image that can manipulate the microparticle [26,30]. In this study, the maximum velocity of rectangular light bars with various widths (e.g., 50, 75, 100, 125, and 150 μm) that can manipulate PS microbeads of different sizes (e.g., 5.8, 10.8, and 15.8 μm in diameter) was measured. Therefore, based on these results, the optimal width and velocity of the rectangular light bars in dynamic light bar arrays I and II (Figure 3) were determined. In addition to the two operation parameters above, the volumetric flow rate of the microchannels was experimentally determined. In this evaluation, microscopic observation was carried out at the sorting and separation zone to examine the stability of the three laminar microflows formed under the tested volumetric flow rate conditions (e.g., 0.1, 0.3, 0.5, and 1.0 μL min^−1^).

### 2.4. Performance Assessment of the Continuous Sorting and Separation of PS Microbeads of Different Sizes

After the operation conditions were determined, the performance of the proposed operation scheme, as illustrated in Figure 2, was assessed experimentally. First, the individual behavior of PS microbeads of the same size was microscopically observed to determine whether their movement course was as described in Figure 2. For this purpose, videos of PS microbeads traveling through the designed dynamic light bar arrays I and II were continuously recorded. In addition, the recovery rate [i.e., (the number of specific-size PS microbeads collected from microchannels I, II, or III)/(the number of total specific-size PS microbeads originally loaded) × 100%] was quantitatively evaluated. After the basic assessment of microbeads of the same size, a mixture of PS microbeads of three different sizes was loaded into the ODEP microfluidic chip. A video of all the microbeads passing through the designed arrays I and II was recorded. Moreover, the purity [i.e., the number of PS microbeads of a specific size collected in the designed microchannel/the number of total PS microbeads collected in the designed microchannel × 100%] of the PS microbeads with diameters of 5.8, 10.8, and 15.8 μm, which were ideally collected in corresponding microchannels III, II, and I, respectively, was evaluated.

### 2.5. Data Presentation

In this study, the experimental data from three separate experiments are presented as the mean ± standard deviation.

## 3. Results

### 3.1. The Technical Features of the Proposed Method for Size-Based Sorting and Separation of Microparticles

Microparticle manipulation based on the ODEP principle has been demonstrated to sort, separate, or purify biological (e.g., cells or bacteria) or nonbiological (e.g., PS microbeads or magnetic microbeads) microparticles based on various properties (e.g., size [26,30], surface antigen [29], or cell viability [28]). Although proof of concept has been successfully demonstrated, the real application of previous demonstrations might be restricted by their batch operation scheme and lack of downstream partitioning and transportation schemes for sample collection. To address this technical issue, this study proposes the utilization of the laminar flow pattern that inherently occurs in a microchannel and ODEP-based microparticle manipulation for this purpose. Briefly, three individual microchannels were designed in the ODEP microfluidic chip. In this work, the three microchannels join only at the central part of the microchannels, defined as the sorting and separation zone. In practice, fluid flows with equal volumetric flow rates were driven in the three individual microchannels to form three laminar flows in the sorting and separation zone. This design is used for continuous sample loading at the beginning of operation as well as for the continuous transport of the sorted and separated microparticles in a partition manner for the final collection. In addition, another technical feature of the proposed method is the use of two dynamic light bar arrays to sort and separate PS microbeads of different sizes in a continuous manner. Overall, based on the abovementioned technical features, size-based sorting and separation of microparticles can be performed in a simple and continuous manner. The proposed continuous operation scheme is not limited to the sorting and separation of the PS microbeads, as in this study; it can also be utilized for the sorting and separation of other biological (e.g., cells or bacteria) or nonbiological microparticles based on differences in their properties.

### 3.2. The Optimal Operation Conditions for the Continuous Sorting and Separation of PS Microbeads

To achieve the operation scheme described in Figure 2, the optimal operation conditions, including the width of the rectangular light bar, the moving velocities of the light bars in dynamic lightweight bar arrays I and II, and the volumetric flow rates of the three microchannels, were experimentally explored. The optimal width of the rectangular light bars (e.g., 50, 75, 100, 125, and 150 μm) and the differences in the maximum velocities of light bars that can manipulate PS microbeads of three different sizes (e.g., 5.8, 10.8, and 15.8 μm in diameter) were first determined experimentally. Figure 3 shows the maximum velocities at which the light bar can manipulate microbeads of three different sizes under various light bar widths. Overall, the maximum velocity at which the light bar could manipulate the microbeads increased with increasing microbead size under the same light bar width conditions (Figure 3). This finding can be explained by the fact that the ODEP manipulation force acting on a manipulated microbead (and thus the measured maximum velocity of the light image that can manipulate the microbead) increases with increasing microbead size, as expressed by Equation (1). Moreover, the measured maximum velocities of the light bar with 150 μm in width that can manipulate the 10.8 and 5.8 μm PS microbeads were relatively uniform (i.e., standard deviation = 0). This phenomenon could be due to the fact that the ODEP manipulation force (and thus the maximum velocity of a light bar that can manipulate microbeads) acting on microbeads was stronger when the light bar with larger width was used. Based on the results in Figure 3, a light bar width of 75 μm was selected, allowing for the maximum difference in the velocity of the light bar that could manipulate the microbeads of any two different sizes to be reached. Under these conditions, the maximum velocities of the light bars that could manipulate 5.8, 10.8, and 15.8 μm diameter microbeads were 16.7 ± 7.8, 60.9 ± 4.3, and 101.9 ± 6.0 μm s^−1^, respectively.

Furthermore, the moving velocity of the five rectangular light bars in dynamic light bar array I was set at 50 μm s^−1^, which was higher than the maximum velocity of the light bar (i.e., 16.7 ± 7.8 μm s^−1^; Figure 3) that could manipulate the smallest microbeads (i.e., 5.8 μm in diameter) in this study. Conversely, the set moving velocity was lower than the maximum velocities of the light bar (i.e., 60.9 ± 4.3 and 101.9 ± 6.0 μm s^−1^, respectively) (Figure 3) that could manipulate the larger microbeads (i.e., 10.8 and 15.8 μm in diameter). This moving velocity setting allowed for dynamic light bar array I to sort and separate larger PS microbeads 10.8 and 15.8 μm in diameter from the 5.8 μm microbeads, as illustrated in Figure 2a,b. In this situation, the smallest microbeads (i.e., 5.8 μm) were continuously transported by the laminar flow in microchannel III and finally collected via the outlet of microchannel III (Figure 2). Conversely, the larger separated microbeads (i.e., 10.8 and 15.8 μm) were further delivered to dynamic light bar array II for secondary sorting and separation via laminar flow in microchannel II [Figure 2b–d]. Similarly, in dynamic light bar array II, the moving velocity of the light bars was set at 95 μm s^−1^, which was greater than the maximum velocity of the light bar (i.e., 60.9 ± 4.3.μm s^−1^; Figure 3) that could manipulate medium-sized microbeads (i.e., 10.8 μm). However, the set moving velocity was lower than the maximum velocity of the light bar (i.e., 101.9 ± 6.0 μm s^−1^; Figure 3) that could manipulate the largest microbeads (i.e., 15.8 μm). This design allowed for the largest microbeads to be sorted and separated from the 10.8 μm microbeads, as illustrated in Figure 2d–f. In this work, three laminar flows were created in the defined sorting and separation zone (Figure 1 and Figure 2). This design is used for continuous sample loading (e.g., PS microbeads of three different sizes in this study) at the beginning of operation as well as for the continuous transport of the sorted and separated microparticles in a partition manner for the final collection, as illustrated in Figure 2. To generate stable laminar flows in the sorting and separation zone, an experimental evaluation was carried out. Briefly, four volumetric flow rates (i.e., 0.1, 0.3, 0.5, and 1.0 μL min^−1^) were set in the three microchannels, followed by microscopic observation in the sorting and separation zone. The results (Supplementary Figure S1) revealed that stable laminar flows could not be generated when the flow rate was 0.1 μL min^−1^ (i.e., the lowest flow rate) with the experimental conditions tested. In this work, a multichannel syringe pump was used to drive fluid flows in the three microchannels. The phenomenon observed at 0.1 μL min^−1^ could be due to the inability of the mechanical pump to perfectly achieve a continuous-flow pattern, particularly at low flow rates. In this work, moreover, the flow rate condition of 1.0 μL min^−1^ might not be appropriate. This is mainly because the residential time of microbeads in the dynamic light bar array was estimated to be 10 s, which is lower than the minimal requirement of 10.5 s (i.e., the calculated time required for a microbead to be initially manipulated and finally released by the light bar). Therefore, the use of a high flow rate of 1.0 μL min^−1^ could affect the performance of the microbead sorting and separation operation. Based on the evaluation, a volumetric flow rate of 0.3 μL min^−1^ was determined, allowing for the flowing microbeads to have more residence time in the sorting and separation zone for more effective sorting and separation via ODEP-based microbead manipulation, as illustrated in Figure 2.

### 3.3. Performance Evaluation of the Proposed Method for the Continuous Sorting and Separation of PS Microbeads of Three Different Sizes

**Figure 4:**
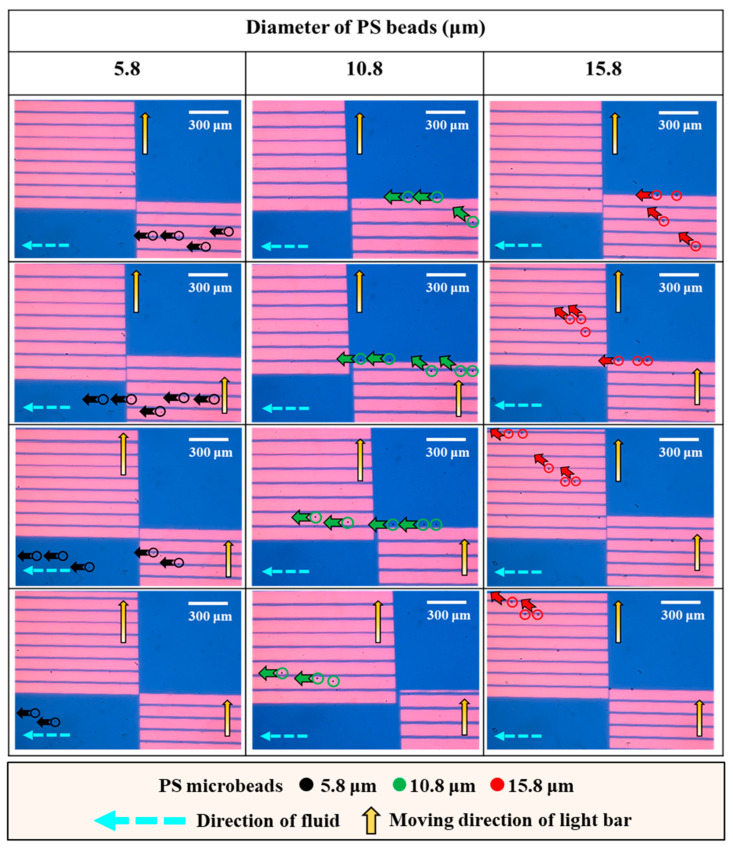
Microscopic photographs showing the movement of PS microbeads (as indicated by black, green, and red arrows for 5.8, 10.8, and 15.8 μm PS microbeads, respectively) of the same size when they traveled through dynamic light bar arrays I and II. The process of moving through the course is shown in a top-down direction for each microbead size.

After the operation conditions were determined, the performance of the proposed operation scheme (Figure 2) for the continuous size-based sorting and separation of PS microbeads was evaluated experimentally. To explore whether the behavior of the microbeads was as illustrated in Figure 2, the motion of PS microbeads of the same size as they traveled through dynamic light bar arrays I and II was first observed via continuous video recording. Figure 4 shows parts of the microscopic photographs from the video recording. For the smallest microbeads (i.e., 5.8 μm), it can be clearly seen from Figure 4 (left column, in a top-down direction) that they were not manipulated by dynamic light bar array I and thus flowed directly through it. The middle-sized particles (i.e., 10.8 μm) were manipulated by dynamic light bar array I and then transported forward to dynamic light bar array II via laminar flow in microchannel II. When reaching dynamic light bar array II, however, they were not further manipulated by the dynamic light bars and thus directly flowed through the array [Figure 4 (the middle column)]. The largest microbeads (i.e., 15.8 μm) were manipulated by both of the dynamic light bar arrays and finally moved forward to the outlet of microchannel I, as expected [Figure 4 (right column)]. In addition to observation of the movement of the PS microbeads, the recovery rate of microbeads of the same size when they passed through the sorting and separation zone was quantitatively evaluated. The results (Supplementary Figure S2) showed that almost 100% of the microbeads 5.8, 10.8, and 15.8 μm in diameter were harvested at the outlets of microchannels III, II, and I, respectively.

**Figure 5:**
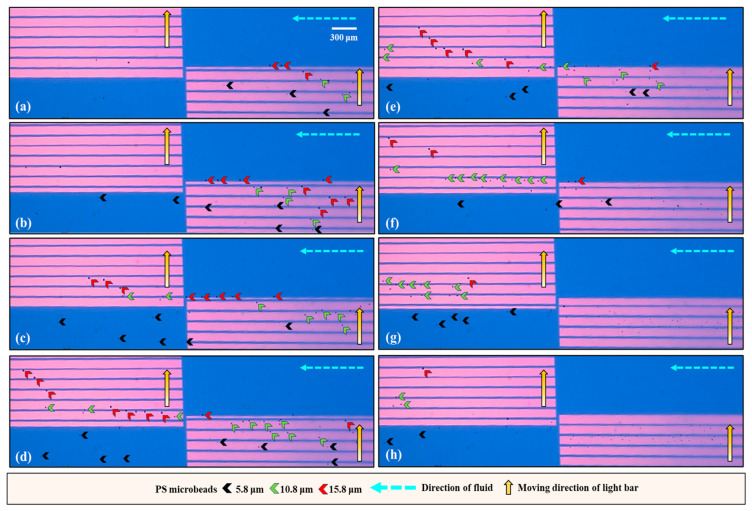
Microscopic photographs [(**a**–**h**) recorded from various timepoints of Video S1] showing the group behavior of PS microbeads of three different sizes (as indicated by the arrows with different colors) when they traveled through the two dynamic light bar arrays.

**Figure 6:**
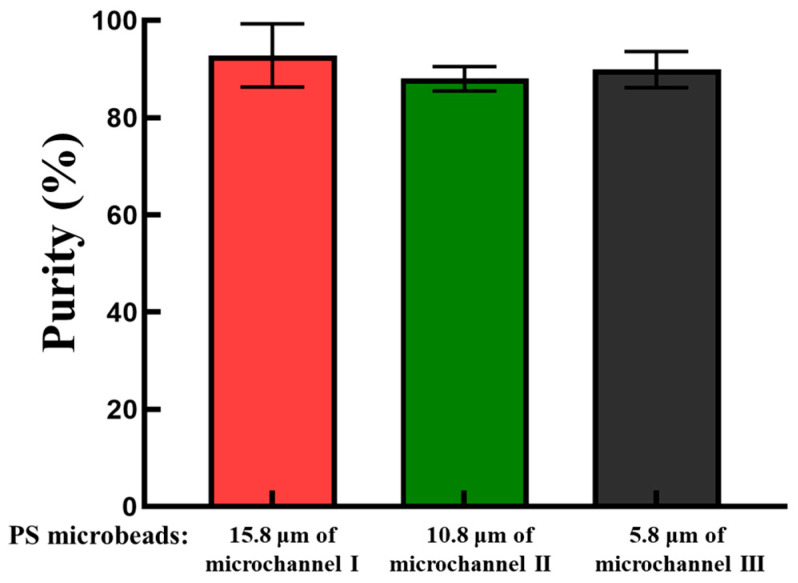
The purities of the 15.8, 10.8, and 5.8 μm PS microbeads collected from the outlets of microchannels I, II, and III, respectively.

After the evaluation based on the abovementioned simple model, the performance of the real application model was further explored. Briefly, a mixture of PS microbeads with three different sizes was loaded into the ODEP microfluidic chip via the inlet of microchannel III, as illustrated in Figure 2a. Again, videos of all microbeads traveling through dynamic light bar arrays I and II were recorded (Supplementary Video S1). Figure 5 shows parts of the microscopic photographs from the video recording. Overall, Figure 5 shows that the group behavior of all microbeads when they traveled through the two dynamic light bar arrays basically followed the operation scheme illustrated in Figure 2. Moreover, in this study, the purities of PS microbeads 5.8, 10.8, and 15.8 μm in diameter that were ideally collected in corresponding microchannels III, II, and I, respectively, were assessed. The results (Figure 6) revealed that the purities of the 5.8, 10.8, and 15.8 μm microbeads collected from the outlets of microchannels III, II, and I were 89.9 ± 3.7, 88.0 ± 2.5, and 92.8 ± 6.5%, respectively. The inability to achieve 100% purity of the harvested microbeads in the outlets of the three microchannels could be partly due to the aggregation of microbeads during the operation process. The alteration of the particle size due to the aggregation of microbeads could therefore affect the performance of the proposed method. This phenomenon could be further improved by continuously loading a microbead sample with a lower density of microbeads. As a proof-of-concept study, this study used microbeads of 5.8, 10.8, and 15.8 μm in diameter for demonstration purposes. Further investigation is required to explore the minimal size difference that can be sorted and separated by the proposed method. As a proof-of-concept demonstration, this study simply used the same flow rate in the three microchannels. The modification of flow rates in the three microchannels holds great potential for various operations. Overall, this study proposes the utilization of the laminar flow pattern inherent to microchannels with ODEP-based microparticle manipulation to achieve size-based sorting, separation, transport, and collection of microparticles in a simple, continuous, and high-performance manner. The proposed continuous operation scheme is not solely for the sorting and separation of the PS microbeads, as in this study; it can also be utilized for the sorting and separation of other biological (e.g., cells or bacteria) or nonbiological microparticles based on differences in their properties.

## 4. Conclusions

The integration of ODEP-based microparticle manipulation in a microfluidic system has been demonstrated for a wide variety of applications; among them, its application for the sorting and separation of microparticles of different sizes has attracted the interest of scientists due to its simplicity, high performance, and ease of operation. Nevertheless, the current operation schemes are normally based on batchwise operation models and lack a downstream operation process for the partitioning, transportation, and collection of sorted and separated microparticles; this could therefore restrict the application of ODEP for high-throughput or continuous size-based microparticle sorting and separation. To address this issue, this study proposes the utilization of the laminar flow regime inherent in a microfluidic channel and ODEP-based microparticle manipulation for this purpose. In this work, an ODEP microfluidic chip encompassing three individual microchannels joining only at the central part of the microchannel was designed and fabricated. In the design, the junction zone of three microchannels was defined as the sorting and separation zone. During operation, fluid flows with equal flow rates were driven in the three individual microchannels to form three laminar flows in the sorting and separation zone. In this zone, two dynamic light bar arrays with different velocities were designed to sort and separate PS microbeads of three different sizes in a continuous-flow manner. The sorted and separated PS microbeads were then continuously transported in three laminar flows in a partition manner for the final collection purpose. In this study, the optimal width (i.e., 75 μm) and velocity of motion of the rectangular light bars in two dynamic light bar arrays (i.e., 50 and 95 μm s^−1^ for arrays I and II, respectively) were explored experimentally for the size-based sorting and separation of PS microbeads. Additionally, the optimal flow rate (i.e., 0.3 μL min^−1^) in the three microchannels was experimentally determined to ensure that the laminar microflows in the sorting and separation zone were stable. The results of the performance evaluation revealed that the proposed method was capable of sorting and separating PS microbeads in a high-purity manner (e.g., the purities of the microbeads were 89.9 ± 3.7, 88.0 ± 2.5, and 92.8 ± 6.5% for the 5.8, 10.8, and 15.8 μm microbeads harvested, respectively). Overall, this study demonstrated that the laminar flow pattern inherently occurs during microchannel and ODEP-based microparticle manipulation to achieve size-based sorting, separation, transport, and collection of microparticles in a simple, continuous, high-performance manner.

## Data Availability

The original contributions presented in the study are included in the article/Supplementary Material, further inquiries can be directed to the corresponding author/s.

## Supplementary Materials

The following supporting information can be downloaded at https://www.mdpi.com/article/10.3390/bios14060297/s1. Figure S1: Microscopic observation of the three laminar microflows formed at the sorting and separation zone under different volumetric flow rate conditions (i.e., 0.1, 0.3, 0.5, and 1.0 μL min^−1^); Figure S2: Evaluation of the recovery rate of microbeads of the same size when they passed through the sorting and separation zone; Video S1: A video clip of three different sizes of PS microbeads traveling through dynamic light bar arrays I and II.
