# Supplementary material for: Combination of an Optically Induced Dielectrophoresis (ODEP) Mechanism and a Laminar Flow Pattern in a Microfluidic System for the Continuous Size-Based Sorting and Separation of Microparticles
Source: Biosensors (Basel). 2024 Jun 6;14(6):297. doi: 10.3390/bios14060297 (PMC11201910; doi:10.3390/bios14060297)
Supplement: Supplementary file 1 [file biosensors-14-00297-s001.zip › supplmentary material_biosensors_P.Y.Chu.pdf]

Article

Combination of an Optically Induced Dielectrophoresis (ODEP) Mechanism and a Laminar Flow Pattern in a Microfluidic System for the Continuous Size-Based Sorting and Separation of Microparticles

Po-Yu Chu [1], Ai-Yun Wu [1], Kun-Yu Tsai [2], Chia-Hsun Hsieh [3,4,5] and Min-Hsien Wu [1,3,4,6,*]

[1] Graduate Institute of Biomedical Engineering, Chang Gung University, Taoyuan City 33302, Taiwan; d000018394@cgu.edu.tw (P.-Y.C.); m1031001@cgu.edu.tw (A.-Y.W.)
[2] Division of Colon and Rectal Surgery, New Taipei Municipal TuCheng Hospital, New Taipei City 23652, Taiwan; edward31705@cgmh.org.tw
[3] Division of Hematology/Oncology, Department of Internal Medicine, Chang Gung Memorial Hospital at Linkou, Taoyuan City 33302, Taiwan; wisdom5000@cgmh.org.tw
[4] Division of Hematology/Oncology, Department of Internal Medicine, New Taipei Municipal Hospital, New Taipei City 23652, Taiwan
[5] College of Medicine, Chang Gung University, Taoyuan City 33302, Taiwan
[6] Department of Biomedical Engineering, Chang Gung University, Taoyuan City 33302, Taiwan
* Correspondence: mhwu@mail.cgu.edu.tw; Tel.: +886-3211-8800 (ext. 3599)

Supplementary Figure

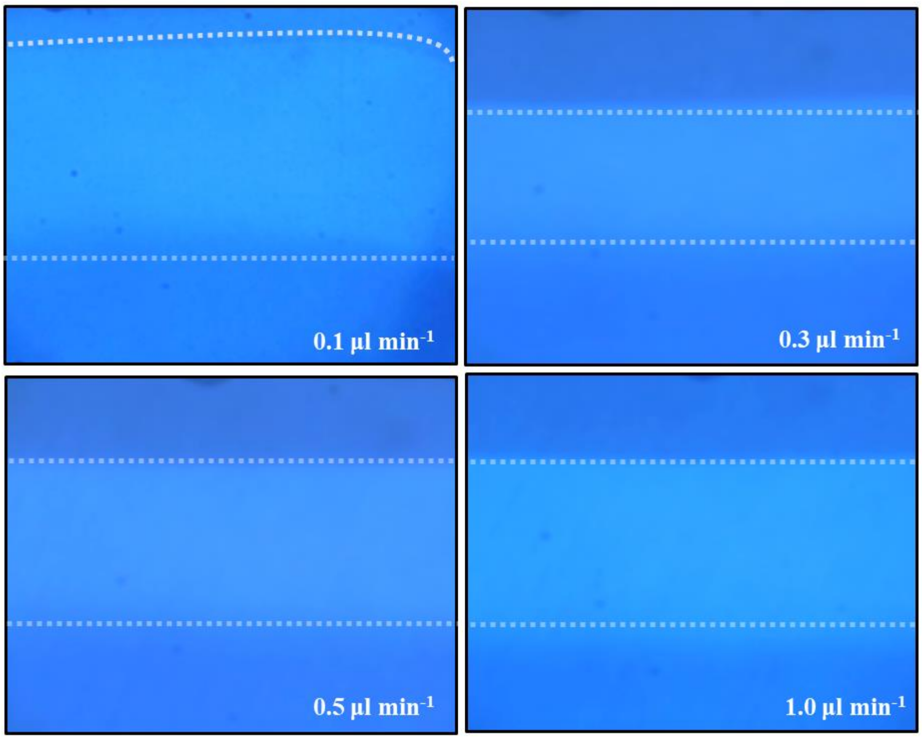

Supplementary Figure S1. Microscopic observation of the three laminar microflows formed at the sorting and separation zone under different volumetric flow rate conditions (i.e., 0.1, 0.3, 0.5, and 1.0 µl min^{-1}).

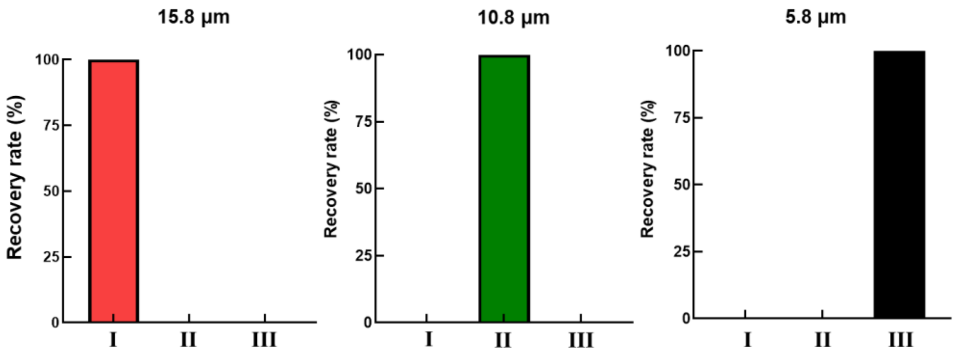

Supplementary Figure S2. Evaluation of the recovery rate of microbeads of the same size when they passed through the sorting and separation zone.

Supplementary Video

Supplementary video clip: A video clip of three different sizes of PS microbeads traveling through dynamic light bar arrays I and II.
